# Chicago Classification of Esophageal Motility Disorders: Lessons Learned

**DOI:** 10.1007/s11894-017-0576-7

**Published:** 2017-07-20

**Authors:** W. O. A. Rohof, A. J. Bredenoord

**Affiliations:** Department of Gastroenterology and Hepatology, Academic Medical Center, Meibergdreef 9, 1105 AZ Amsterdam, the Netherlands

**Keywords:** Esophageal motility, Manometry, Chicago classification, Achalasia, Esophageal spasm

## Abstract

**Purpose of Review:**

High-resolution manometry (HRM) is increasingly performed worldwide, to study esophageal motility. The Chicago classification is subsequently applied to interpret the manometric findings and facilitate a diagnosis of esophageal motility disorders. This review will discuss new insights regarding the diagnosis and management using the Chicago classification.

**Recent Findings:**

Recent studies have demonstrated that high-resolution manometry is superior to conventional manometry, and has a higher sensitivity to diagnose achalasia. Furthermore, the subclassification of achalasia as used in the Chicago classification has prognostic value and can be used to direct treatment. Diagnosis of esophageal spasm has been improved by using the distal latency as diagnostic criterion. Recently, criteria for minor disorders of peristalsis have been sharpened, leading to a lower rate of patients with abnormal results, thereby increasing the relevance of a diagnosis.

**Summary:**

High-resolution manometry is now considered the gold standard for diagnosis of esophageal motility disorders. The Chicago classification provides a standardized approach for analysis and categorization of abnormalities that has led to a significant increase in our knowledge regarding the diagnosis and management of motility disorders. Further refinement of the classification will be required.

## Introduction

When a patient is referred with dysphagia, an upper endoscopy is the essential first step to exclude structural abnormalities such as esophageal carcinoma, stricture, or eosinophilic esophagitis. If no abnormalities are found, esophageal manometry is usually the next step for the detection of esophageal motility disorders.

**Fig. 1:**
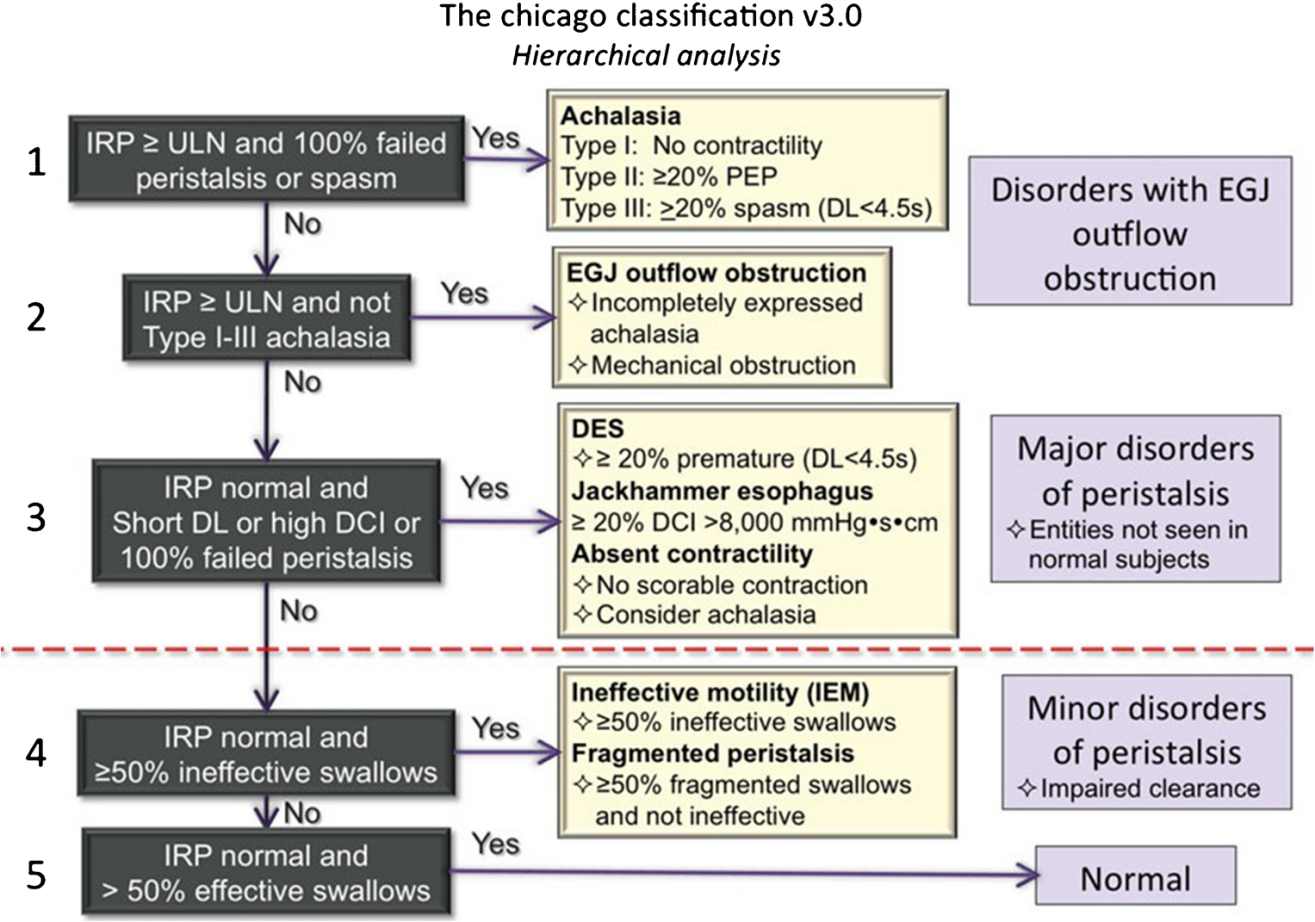
The Chicago classification is a hierarchical classification scheme. The initial step is to evaluate the relaxation of the esophagogastric junction upon swallowing by using the IRP. If elevated, patients should be classified as having achalasia or EGJ outflow obstruction, depending on the peristalsis. In case of a normal IRP, peristalsis is classified based on absence, distal latency, DCI, and fragmentation. If there are abnormalities, patients are classified as having a major or minor disorder of peristalsis. Major disorders are never observed in controls, in contrast to minor disorders. If a patient has a normal IRP and more than 50% of swallows are effective, esophageal motility is normal. Reprinted with permission from John Wiley and Sons

**Fig. 2:**
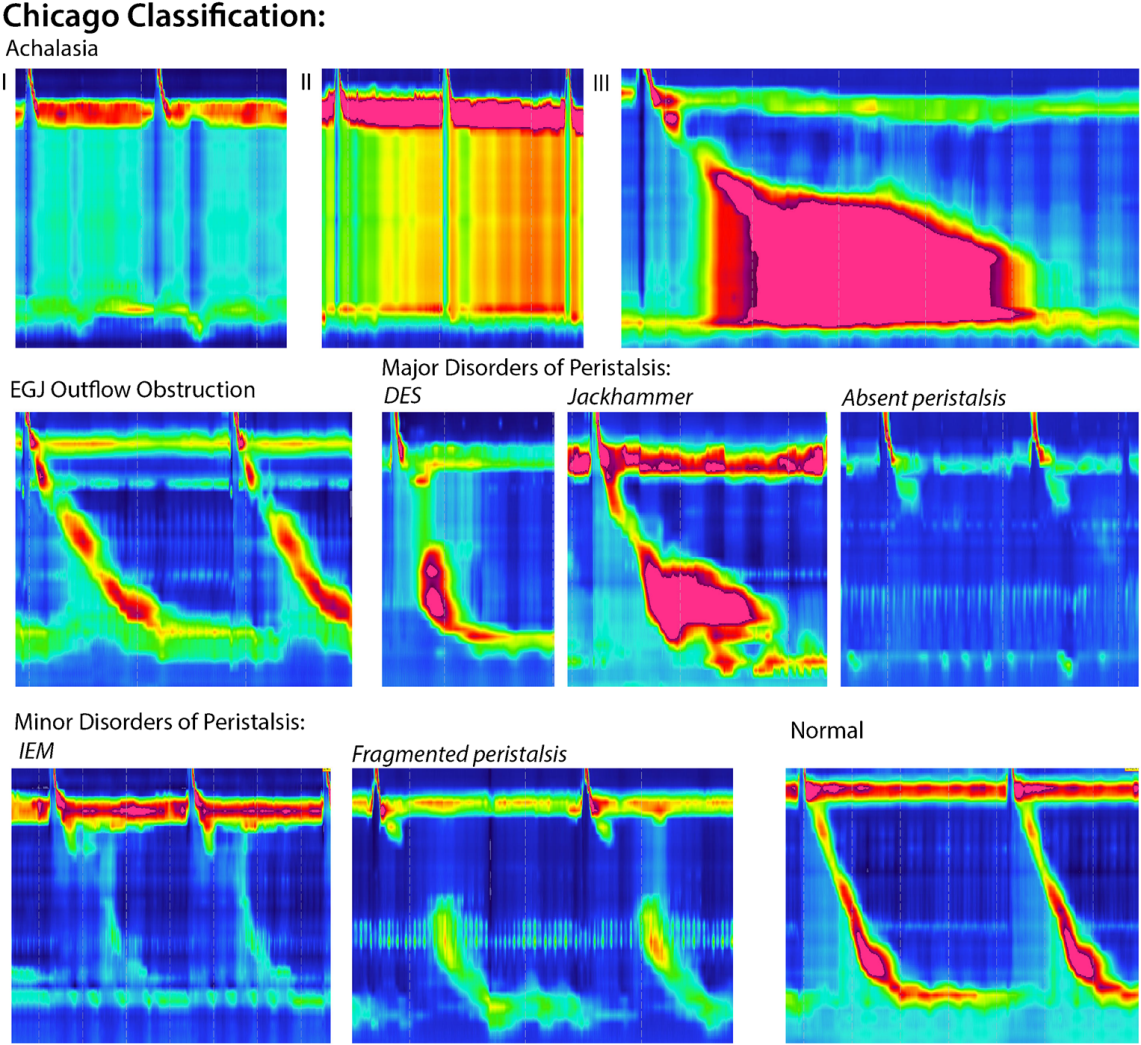
In this figure, examples of high manometry color plots are shown of the individual disorders as classified in the Chicago classification

In the last few years, there have been significant improvements in the manometric sensor technology, manometric data display, and manometric data analysis. High-resolution manometry (HRM) provides an improved and more detailed information on esophageal motility when compared to conventional manometry, and today is considered the best test for diagnosis of motility disorders. In this technique, 20–36 pressure sensors are spaced at 1 cm intervals, decreasing the spacing between the sensors significantly compared to conventional manometry (usually 3–6). To overview all these pressure signals, color plots are utilized, also referred to as pressure topography, enabling quick and intuitional interpretation. To enable objective analysis of HRM metrics and topography, a classification scheme has been developed, namely the Chicago classification [1] (Figs. 1 and 2).

This hierarchical system of analysis has four major categories that are classified based on LES relaxation and motility of esophageal body: (1) incomplete LES relaxation (achalasia or esophagogastric junction outflow obstruction), (2) major motility disorders (absent contractility, distal esophageal spasm, and hypercontractile or jackhammer esophagus), (3) minor motility disorders (ineffective esophageal motility or fragmented peristalsis), and (4) normal esophageal motility [1].

Using the Chicago classification, HRM not only has superior inter-rater agreement [2, 3] and is easier to learn [4], but also has higher diagnostic accuracy, with a significantly lower chance of an incorrect diagnosis compared to conventional manometry, both for experts and non-experts. [3] In addition, after a 6-month follow up period, diagnoses are more frequently confirmed using HRM than using conventional manometry (89 vs 81%, *P* = 0.07) [5••]. Therefore, HRM is considered the gold standard for diagnosis of esophageal motility disorders.

The widespread use of HRM in combination with the Chicago classification in clinical studies and common practice has provided considerable insights regarding esophageal motility disorders in recent years. In this review, the impact of this technology and Chicago classification on clinical studies and on clinical practice both current and in the future will be discussed.

## Achalasia

Achalasia is a disorder characterized by the absence of peristalsis and a defective relaxation of the LES, resulting in impaired bolus transport and stasis of food in the esophagus. In the Chicago classification, a diagnosis of achalasia is based on an elevated median integrated relaxation pressure (IRP) in combination with failed peristalsis or spasm [1]. In a recent trial by Roman et al., 245 patients with unexplained dysphagia were randomized to undergo conventional manometry or HRM. Interestingly, the detection rate of achalasia by HRM was significantly higher (26 vs 12%, *P* < 0.05) [5••]. In contrast, the rate of non-specific motility disorders was lower in the HRM group compared to the conventional manometry group (3 vs 12%, *P* < 0.05).

The use of HRM has led to the subclassification of achalasia into three clinically relevant subclasses based on the pattern of contractility in the esophageal body: in type I, no pressure waves are recorded in the distal esophagus; type II is characterized by panesophageal pressurizations; whereas in type III, at least 20% of swallows reveal rapidly propagating or spastic simultaneous contractions. Importantly, treatment outcome depends on this new subclassification, and therefore the subtype can be used to predict treatment response: All studies uniformly observed that the best treatment response was seen in patients with type II achalasia (95–96%) and the worst response in patients with type III achalasia (29–70%) [6–9]. The subclassification is relevant, especially for patients with type III achalasia, since the success rate of treatment is lower for pneumatic dilation compared to laparoscopic Heller myotomy in a randomized trial (40 vs 86%) [8]. Recently, peroral endoscopic myotomy (POEM) has been described with high success rates. In this technique, the endoscopist creates a submucosal tunnel to dissect the circular muscle. In patients with type III achalasia, the length of the myotomy can be adjusted to the length of the spastic segment in the distal esophagus as observed on HRM. Of interest, the 1-year success rate of POEM has been reported to be 90%, even in patients with type III achalasia [10]. Results of randomized controlled trials comparing POEM to LHM and pneumatic dilation are currently awaited.

## EGJ Outflow Obstruction

Esophagogastric junction (EGJ) outflow obstruction is a manometric diagnosis, defined as an incomplete LES relaxation (elevated median IRP) together with an intact or weak peristalsis. It is rather rare but increasingly diagnosed due to the increased use of HRM. Recently, two case series have been published both focusing on the characterization and follow up of this condition [11, 12]. The symptoms most often reported are dysphagia, heartburn, and retrosternal pain, the same symptoms as for which HRM is usually performed. It was often not clear whether the patients’ symptoms were related to the manometric abnormality of a high IRP and the high IRP is sometimes regarded not clinically significant. Stasis was described in only 28 to 38% of patients, using either barium swallows or impedance monitoring. In 20–40%, patients’ symptoms resolved without intervention, mostly during the first 6 months of follow up. Therefore, a waiting period before initiating treatment is recommended. If treatment is considered, Botox injections should be attempted first, because of reported success in retrospective uncontrolled studies (58–100% success rate, 3 studies, 47 patients), although the effect is usually short-lived [11–13]. Interestingly, only a small minority of patients progresses to achalasia [11].

## Distal Esophageal Spasm

Esophageal spasm is considered to be the consequence of impaired inhibitory innervation and results in symptoms of dysphagia and retrosternal pain. In the latest Chicago classification (version 3.0), distal esophageal spasm is defined as the occurrence of premature contractions in at least 20% of swallows in conjunction with normal EGJ relaxation [1]. A premature contraction is defined as a swallow with a distal latency (time from onset of the UES relaxation to contractile deceleration point), of less than 4.5 s. Before the Chicago classification, esophageal spasm was diagnosed on the basis of simultaneous or rapidly propagated contractions [14]. This is however a non-specific parameter observed in up to 8.0% of patients undergoing HRM, not only in patients with esophageal spasm, but also in patients with weak peristalsis, outflow obstruction and hypertensive peristalsis [15]. Distal latency (observed in 2.2% of patients) has a better correlation with symptoms and most patients with a distal latency of <4.5 s are indeed eventually diagnosed with distal esophageal spasm or achalasia (type III, spastic type) [15]. There is evidence to suggest that patients with rapid contractions without a short distal latency represent a group of patients with spastic motor disorders with a milder phenotype [16].

Treatment should be aimed at reducing the spastic contractions in the distal esophagus. For this purpose, calcium antagonists and nitrates have been used. Small controlled studies show a decrease in chest pain scores but no change in dysphagia scores for both treatment options [17, 18]. Botulinum toxin injection in the esophageal body causes muscle paralysis by blocking acetylcholine. In a randomized controlled trial, botulinum toxin injection was superior to a sham procedure to relieve symptoms (50 vs 10% success) [19•]. Finally, POEM is a relatively new treatment option, with a high reported success rate (87% (CI 78–93%)) in a recent meta-analysis of case series [20]. The rate and severity of adverse effects is very acceptable. Compared to botulinum toxin injection, POEM has the advantage of sustained long-benefit, but it should be noted that this has not been tested in a randomized matter and long-term outcome data are lacking.

## Jackhammer Esophagus

Jackhammer esophagus is a hypercontractile motility disorder observed in 4.1% of patients that undergo manometry [21]. Isolated hypercontractility following a single swallow in the esophagus is also observed in healthy controls and is usually not associated with symptoms [22]. Therefore, a new parameter was introduced to measure the vigor of peristalsis, the distal contractile integral (DCI). This is a multiplication of length, duration, and amplitude of contractions. Asymptomatic controls never have a DCI over 8000 mmHg cm s, and therefore ≥20% of swallows with a DCI >8000 mmHg cm s is required for a diagnosis of jackhammer esophagus.

The relationship between symptoms and hypercontractility is not always straightforward. In a recent study of 34 patients with jackhammer esophagus, most patients reported dysphagia (68%) and/or chest pain (47%) [23]. The symptom of dysphagia is accompanied with strong contractions of the LES, signs of a possible outflow obstruction, and a very high DCI. The presence of a multipeaked contraction seems to be of limited relevance.

Treatment options for jackhammer esophagus include botulinum injection and POEM, although data are scarce. Marjoux et al. reported a success rate of 71% for botulinum injection after 2 months in a retrospective case series [24]. This is confirmed in the randomized controlled trial by Vanuytsel et al., in which 7 of 22 patients had a jackhammer esophagus, although no specific results for his subgroup are reported [19]. Five studies have evaluated the success of POEM in patients with jackhammer esophagus. A recent meta-analysis of the studies including 37 patients showed a success rate of 69% (CI 53–81%), which is significantly lower than in patients with achalasia (*P* = 0.01) [20].

## Absent Contractility

When no scorable contraction but a normal IRP is observed, patients are classified as having absent contractility. This motility pattern is classically seen in patients with systemic scleroderma and is caused by myopathy of the smooth muscle. Absent esophageal peristalsis is however not a universal finding among patients with systemic scleroderma. In a large prospective study of 200 patients with scleroderma, absent contractility (56%) was the most frequent diagnosis, followed by normal motility (26%) and ineffective esophageal motility (10%) [25]. Absent contractility is associated with longer disease duration, interstitial lung disease, higher gastrointestinal symptom scores, and impaired quality of life [25]. The myopathy not only affects the esophageal body, but also the esophageal sphincter predisposing to frequent and long reflux episodes. This is reflected by the high rate of reflux symptoms (>80%), use of acid suppression (92%), and esophagitis (31%) [25, 26]. Multiple rapid swallows, a simple provocation test, is abnormal in 82% of patients, and could potentially be used to detect esophageal involvement [26]. Future studies will clarify whether this provocation test should be performed in standard practice.

Treatment options for absent peristalsis are limited: No pharmacological intervention can restore esophageal peristalsis. Reflux symptoms should be treated by acid suppression, and dietary and lifestyle interventions have been proposed. Fundoplication or gastric bypass surgery is absolutely contraindicated in the patients and should be avoided.

## Minor Disorders of Peristalsis: Ineffective Motility and Fragmented Peristalsis

Ineffective esophageal motility is diagnosed when >50% of swallows is ineffective, that is either failed (DCI <100mmHg cm s) or weak (DCI 100–450 mmHg cm s). Fragmented peristalsis is defined as >50% of swallows with a large break (>5 cm) and not matching criteria for ineffective esophageal motility [1].

In the previous HRM classification system, criteria for a minor disorder of peristalsis were less stringent. As a result, a minor disorder was also frequently observed in healthy volunteers. In patients, a minor disorder of peristalsis was poorly associated with symptoms or esophageal stasis. Moreover, long-term follow up of patients with minor disorders revealed that the vast majority of patients never developed severe symptoms in the 6.4 years that they were followed and that more than 70% of the patients even became completely asymptomatic [27•].

To increase the clinical significance, the Chicago classification for minor disorders of peristalsis is adjusted, and several conditions that were previously seen as a disorder are now relabeled as normal. In a recent study by Monrroy et al., the latest version of the Chicago classification was directly compared to the previous version. In asymptomatic controls, minor disorders were diagnosed significantly less often using the latest version (15 vs 25%), and esophageal manometries were significantly more often classified as normal (77 vs 64%) [28]. Likewise, the use of the latest classification in symptomatic patients resulted in less diagnosis of minor disorders (33 vs 25%), and increased the rate of normal findings (50 vs 42%). As the relevance of minor disorders is questionable, the authors conclude that the latest classification increases the relevance of abnormal results; however, it should be noted that for minor disorders of esophageal motor disorders, the clinical significance remains unclear.

## Discussion

Today, high-resolution manometry is considered the gold standard for diagnosis of esophageal motility disorders. The development and ongoing improvement of the Chicago classification facilitates a standardized diagnosis and provides opportunities for a standardized management of motility disorders.

As demonstrated in this review, the Chicago classification has driven research in the esophageal field globally and contributed to a sharp increase of knowledge in the recent years. The classification provides a basis to further increase the insight in the pathophysiology of the disorders, which has remained unclear for the larger part. Moreover, with the current standardized classification scheme, new outcome studies—preferably randomized trials—can further clarify the best treatment options. Since most esophageal motility disorders are rare, randomized trials have to be performed in multiple centers for adequate power. The Chicago classification is indispensable for the design and planning of these studies.

Further refinement of the classification will be required. For example, UES abnormalities and post-surgical problems are not classified now, while HRM is very well capable of imaging these abnormalities.
